# Effect of Antioxidants and Apoptosis Inhibitors on Cryopreservation of Murine Germ Cells Enriched for Spermatogonial Stem Cells

**DOI:** 10.1371/journal.pone.0161372

**Published:** 2016-08-22

**Authors:** Seung-Jung Ha, Byung-Gak Kim, Yong-An Lee, Yong-Hee Kim, Bang-Jin Kim, Sang-Eun Jung, Myeong-Geol Pang, Buom-Yong Ryu

**Affiliations:** 1 Department of Animal Science and Technology, Chung-Ang University, Anseong, Gyeonggi-Do, Republic of Korea; 2 Bio Environment Technology Research Institute, Chung-Ang University, Anseong, Gyeonggi-Do, Republic of Korea; 3 Laboratory of Bioimaging Probe Development, Singapore Bioimaging Consortium, Agency for Science, Technology and Research, Singapore; EFS, FRANCE

## Abstract

Spermatogonial stem cells (SSCs) are germline stem cells that serve as the foundation of spermatogenesis to maintain fertility throughout a male’s lifetime. To treat male infertility using stem cell banking systems and transplantation, it is important to be able to preserve SSCs for long periods of time. Therefore, this study was conducted to develop an optimal cryopreservation protocol for SSCs using antioxidants and apoptosis inhibitors in freezing medium. No differences were observed compared to controls when SSCs were cryopreserved in the presence of apoptosis inhibitors by themselves. However, mouse germ cells cryopreserved in basal medium containing the antioxidant hypotaurine (14 mM) resulted in significantly greater proliferation potential and mitochondrial activity. Furthermore, treatment groups with combinations containing 200 mM trehalose and 14 mM hypotaurine showed higher proliferation rates compared to controls. In addition, several serum free conditions were evaluated for SSC cryopreservation. Treatment media containing 10% or 20% knockout serum replacement resulted in similar cryopreservation results compared to media containing FBS. SSC transplantation was also performed to confirm the functionality of SSCs frozen in 14 mM hypotaurine. Donor SSCs formed normal spermatogenic colonies and sperm in the recipient testis. These data indicate that inclusion of 14 mM hypotaurine in cryopreservation media is an effective way to efficiently cryopreserve germ cells enriched for SSCs and that knockout serum replacement can replace FBS in germ cell cryopreservation media.

## Introduction

Spermatogonial stem cells (SSCs) are adult germline stem cells that serve as the foundation of spermatogenesis throughout the lifetime of a male. SSCs ultimately differentiate into sperm that deliver paternal genetic information to the next generation [1]. The SSC population is able to perform these roles because of an intricate balance in fate decisions between self-renewal and differentiation, resulting in constant numbers of undifferentiated SSCs and differentiating germ cells committed to become sperm. [2, 3]. Because of the role of SSCs in perpetuation of a male’s germline and their susceptibility to death after chemotherapy to treat cancer, considerable effort has been placed on developing techniques for their long-term preservation. These techniques include SSC culture, SSC transplantation, and SSC cryopreservation [4].

Previous work has suggested that SSCs can be cryopreserved [2, 5–9] and that a combination of slow freezing followed by rapid thawing is an effective way to preserve these valuable cells [9, 10]. Furthermore, this method of preservation, as opposed to long-term culture or germline maintenance via xenotransplantation, may be clinically useful for humans because of its convenience. Additionally, evidence from mouse experiments shows that thawed SSCs retain the ability to successfully recolonize infertile mouse testes, as demonstrated by the recipient mouse’s ability to naturally sire offspring. Importantly, offspring from cryopreserved SSCs, do not exhibit genetic or epigenetic errors [11–14]. Although previous reports of SSC cryopreservation have demonstrated the functional capacity of thawed SSCs to give rise to offspring, the efficiency of recovery of functional SSCs after thawing is less than ideal. The process of cryopreservation leads to cryoinjuries that disrupt the normal biological function of cells. These disruptions include mitochondrial dysfunction, DNA fragmentation, oxidative stress, osmotic stress, and induction of apoptosis. To minimize these deleterious disruptions, it is essential to include cryoprotective agents in any cryopreservation medium. These agents can be broadly defined as either permeable cryoprotective agents (PCAs) or additive cryoprotective agents (ACAs) according to their particular mode of cryoprotection [13, 15–17]. Interestingly, previous studies have demonstrated that dimethyl sulfoxide (DMSO) was the most effective PCA for murine SSCs [13].

The aim of the current study was to evaluate several ACAs for their cryoprotective efficacy for the freezing of murine germ cells enriched for SSCs. Specific classes of ACAs of interest in the study include both antioxidants and apoptosis inhibitors that will respectively reduce oxidative stress and apoptosis, arguably the main contributors to poor cryopreservation efficiency of SSCs. Although, these chemicals have not actively been evaluated for the cryopreservation of SSCs, their value in the cryopreservation of other mammalian cells and tissues such as sperm, embryos, hematopoietic stem and progenitor cells, and hepatocytes has been extensively demonstrated [18–24]. Thus, the objective of this work was to determine the efficacy of adding antioxidants (ascorbic acid, glutathione, hypotaurine, glutathione peroxidase, and catalase) or apoptosis inhibitors (Z-VAD-fmk and Y-27632) to SSC cryopreservation media. Efficacy was determined based on observations of post-thaw viability, recovery, mitochondrial activity, *in vitro* proliferation capacity and ability to re-establish spermatogenesis in recipient testes.

## Materials and Methods

### Isolation and culture of germ cells

Unless otherwise stated, all reagents were purchased from Sigma-Aldrich. Animal procedures were approved by the Animal Care and Use Committee of Chung-Ang University (permit number 13–0001) in accordance with the *Guide for the Care and Use of Laboratory Animals of the National Institutes of Health*. Donor mice were 6- to 8-day-old C57BL/6-TgEGFP (C57GFP; Jackson Laboratory) pups that expressed the enhanced green fluorescent protein (eGFP). Recipient mice for all germ cell transplantation experiments were C57BL/6 mice. All donor and recipient mice were sacrificed by CO_2_ inhalation.

Isolation and culture of testis cells enriched for SSCs was performed as previously reported with slight modification [25]. Briefly, testes were collected, washed in Dulbecco's phosphate-buffered saline (DPBS; Invitrogen), and decapsulated. To generate single cell suspensions, testes were digested at 37°C for 5 minutes with trypsin-ethylenediaminetetraacetic acid (Invitrogen; 0.25%) and DNAse I (Roche; 7.0 mg/mL) in DPBS. After initial digestion, seminiferous tubules were dispersed and incubated for an additional 5 minutes at room temperature. Digested cells were suspended in isolation medium consisting of Dulbecco's modified Eagle medium (Invitrogen) containing fetal bovine serum (10%), L-glutamine (2 mM), b-mercaptoethanol (0.1 mM), penicillin (100 U/mL), and streptomycin (100 mg/mL), and filtered through a nylon mesh with 40 μm pores (BD Biosciences). Filtered cells were only used for subsequent experiments if viability was determined to be greater than 95% by trypan blue exclusion. Following viability analyses, single cell suspensions were centrifuged at 600 x g at 4°C for 7 minutes and cells were resuspended at a concentration of 10 × 10^6^ cells/mL in the isolation medium. To remove cellular debris and erythrocytes, 2 mL of the cell suspension was overlaid on 2 mL of 30% Percoll and centrifuged at 600 x g for 10 minutes at 4°C. Following Percoll centrifugation, SSCs were enriched using magnetic activated cell sorting (MACS) and cultured for 6 weeks, as previously described [26]. Briefly, after Percoll separation, testis cells were resuspended and labeled for MACS using anti-Thy-1 microbeads (Miltenyi Biotech) [27]. Following MACS isolation, 0.1 × 10^6^ Thy-1 positive cells were placed per well in 12-well culture plates containing mitotically inactivated SIM mouse embryo-derived thioguanine- and ouabain-resistant feeder cells to initiate cultures of testis cells enriched for SSCs. SSC cultures were maintained in mouse serum-free medium containing 1 ng/mL basic fibroblast growth factor (R&D Systems), 10 ng/mL glial-derived neurotrophic factor (R&D Systems), and 75 ng/mL glial-derived neurotrophic factor family receptor alpha 1 (GFRα-1; R&D Systems), as previously described [27]. Culture medium was replaced every 2 to 3 days and cell cultures were passaged 1:2 or 1:3 weekly.

### Cryopreservation

After culture, single cells, recovered by trypsinization, were suspended at 2.5 × 10^5^ cells/mL of freezing medium and placed in 1.8-mL cryovials (Corning). Cryovials were frozen in a Nalgene freezing container at a rate of -1°C per minute to -80°C and stored overnight at -80°C. After overnight storage, cryovials were placed in liquid nitrogen for long-term storage (at least one month). Cryopreservation stock medium, hereafter referred to as basal freezing medium, consisted of DPBS containing 10% fetal bovine serum (FBS) and10% DMSO (v/v). Treatment media was generated by mixing aqueous solutions (containing double the final concentration of cryoprotectant in DPBS) 1:1 with freezing medium containing 20% FBS and 20% DMSO resulting in treatment media containing the appropriate concentration of cryoprotectant in basal freezing medium (10% FBS and 10% DMSO). Cryopreservation agents, included five antioxidants, ascorbic acid (0.1, 0.5, 1 mM), glutathione (50, 100, 200 μM), hypotaurine (3.5, 7, 14 mM), glutathione peroxidase (1, 5, 10 U/mL), catalase (50, 100, 200 μg/mL), and two apoptosis inhibitors, Benzyloxycarbonyl-Val-Ala-DL-Asp-fluromethylketone (Z-VAD-fmk; 15, 30, 60 μM) and Trans-f-[(1R)-aminoethyl]-N-4-pyridinyl cyclohex anecar–boxamide dihydrochloride (Y-27632; 50, 100, 200 μM).

### Viability and proliferation analysis after freeze-thawing

To compare the efficiency between cryopreservation media after 1 month of cryopreservation, recovery and proliferation capacity of thawed cells were determined. Frozen cells were thawed at 37°C for 2.5 minutes. After thawing, the cell suspension was diluted to 5 mL using MEM alpha containing 10% FBS in a drop-wise manner. Survival rate was determined using trypan blue exclusion. To determine proliferation capacity, thawed germ cells were cultured in mouse serum-free medium for 1 week as described above. After culture, the cells were dissociated from culture plates using trypsin digestion and eGFP expressing cells were quantified using fluorescent microscopy. Germ cells that were frozen in cryopreservation media without antioxidants or apoptosis inhibitors were used as control groups. The recovery rate and proliferation capacity of thawed germ cells were determined using the following equations [28]:

$$\text{Recovery rate }\left(\text{\%}\right)\;=\text{ Number of recovered viable cells after freeze},\text{ thaw},\text{ and washing }\times \;100\;/\text{ Number of cells frozen }\left(2.5\;\times \;10^{5}\text{cells}\right)$$

Proliferation capacity (%) = Number of cells recovered after freeze, thaw, and culture × 100 / Number of cells recovered from the control group after freeze, thaw, and culture.

### Immunocytochemistry

Germ cell purity in single cell suspensions was determined using immunofluorescence staining for VASA homolog (VASA). To quantify undifferentiated spermatogonia, cells were stained for promyelocytic leukemia zinc finger (PLZF), and GFRα1. To label cells, 20 μL of single cell suspension (1 × 10^6^ cells/mL) were plated per well of a Teflon-printed slide (12-wells/slide, 5 mm diameter/well; Electron Microscopy Sciences) and incubated for 10 minutes at 37°C. After cells were attached, they were fixed with 4% paraformaldehyde for 30 minutes and treated with 0.1% Triton X-100 in DPBS at room temperature to increase cell permeability. Cells were then blocked for 30 minutes in DPBS containing 5% bovine serum albumin and incubated with primary antibodies over night at 4°C. The primary antibodies used for immunostaining included mouse anti-human PLZF (Calbiochem), rabbit anti-human VASA (Abcam), and rabbit anti-human GFRα1 (Abcam) diluted 1:200. Following incubation with primary antibodies, cells were washed three times with DPBS and incubated with secondary antibodies, diluted 1:200, for 1 hour at room temperature. Secondary antibodies used for immunostaining included Alexa fluor 568- conjugated goat anti-mouse IgG (Invitrogen) and TRITC-conjugated goat anti-rabbit IgG (Jackson ImmunoResearch). Following incubation with secondary antibodies, cells were washed three times in DPBS and mounted with VectaShield mounting media containing 4’, 6-diamidino-2-phenylindole (DAPI; Vector Laboratories). Cells were analyzed under a Nikon TS-1000 microscope with NIS Elements imaging software. The percentages of PLZF-, VASA-, and GFRα1-positive cells were determined in five randomly selected microscopic fields by dividing the number of labeled cells by the total number of GFP-expressing cells. Negative controls were generated by incubation with DPBS containing 5% bovine serum albumin rather than primary antibodies.

### Analysis of mitochondrial activity after cryopreservation

Following thawing, cryopreserved cells were analyzed for mitochondrial activity using the CellTiter-Glo^®^ luminescence assay (Promega) for measurement of adenosine triphosphate (ATP), as instructed. Briefly, cells (0.05 × 10^6^ cells/well) were seeded onto wells of 96-well plates and cultured for 30 minutes. After attachment, cells were lysed and luminescence produced from an ATP-mediated chemical reaction was determined using a GloMax-Multi+ Detection System. The measured amount of ATP is reported as a percentage of the relative luminescence unit (RLU) value for cells cryopreserved in treatment media against the RLU value of cells cryopreserved in basal freezing media [(RLU value for each treatment group / control RLU value) × 100].

### Transplantation

Although proliferation capacity is a useful analysis of post-thaw cellular function, it does not definitively evaluate post-thaw SSC function. Thus, to quantify post-thaw SSC viability and function directly, the germ cell transplantation technique was performed. For all transplantation experiments, C57BL/6 mice were used as recipients. Six to eight weeks prior to transplantation, 6-week-old recipient mice were treated with busulfan (44 mg/kg) to deplete endogenous spermatogenesis. One month after freezing, germ cells were thawed and cultured for 1 week. Recipient mice were intraperitoneally anesthetized with ketamine (75 mg/kg) and medetomidine (0.5 mg/kg), and germ cells enriched for SSCs were transplanted into their testes. Approximately 8 μL (2.5 × 10^6^ cells/mL) of donor cells were injected into each recipient testis through efferent ducts, resulting in the filling of approximately 80% of seminiferous tubules, as previously described [29]. Two months after transplantation, recipient mice were euthanized and testes were collected and decapsulated. The seminiferous tubules were gently dispersed and analyzed using fluorescence microscopy for the presence of eGFP positive donor colonies that were ≥1 mm in length, as previously described [30]. Colony numbers were determined as the number of colonies per 10^5^ transplanted cells (Colonies / 10^5^ cells transplanted = Number of colonies × 10^5^ / Number of cells transplanted), as previously described [28]. To account for the effect of post thaw activity, colony numbers were then normalized as the number of total cells recovered after culture (Colonies / Total number of cells cultured = Number of colonies × Total number of cells cultured / Number of cells transplanted), as previously described [28]. To verify completion of donor-derived spermatogenesis in recipient testes, additional testes were collected for histological analysis 2–3 months after transplantation.

### Statistical Analysis

All data (values presented as means ± SEM) were analyzed by analysis of variance (ANOVA) using SPSS version 18 software. Means were compared using Tukey’s honestly significant difference test and the differences were considered significant if *P* < 0.05. In initial experiments, analyses of different concentrations of a specific cryoprotectant were conducted independently, each with its own control group.

## Results

### Effects of antioxidants and apoptosis inhibitors on cryopreservation of germ cells enriched for SSCs

**Fig 1:**
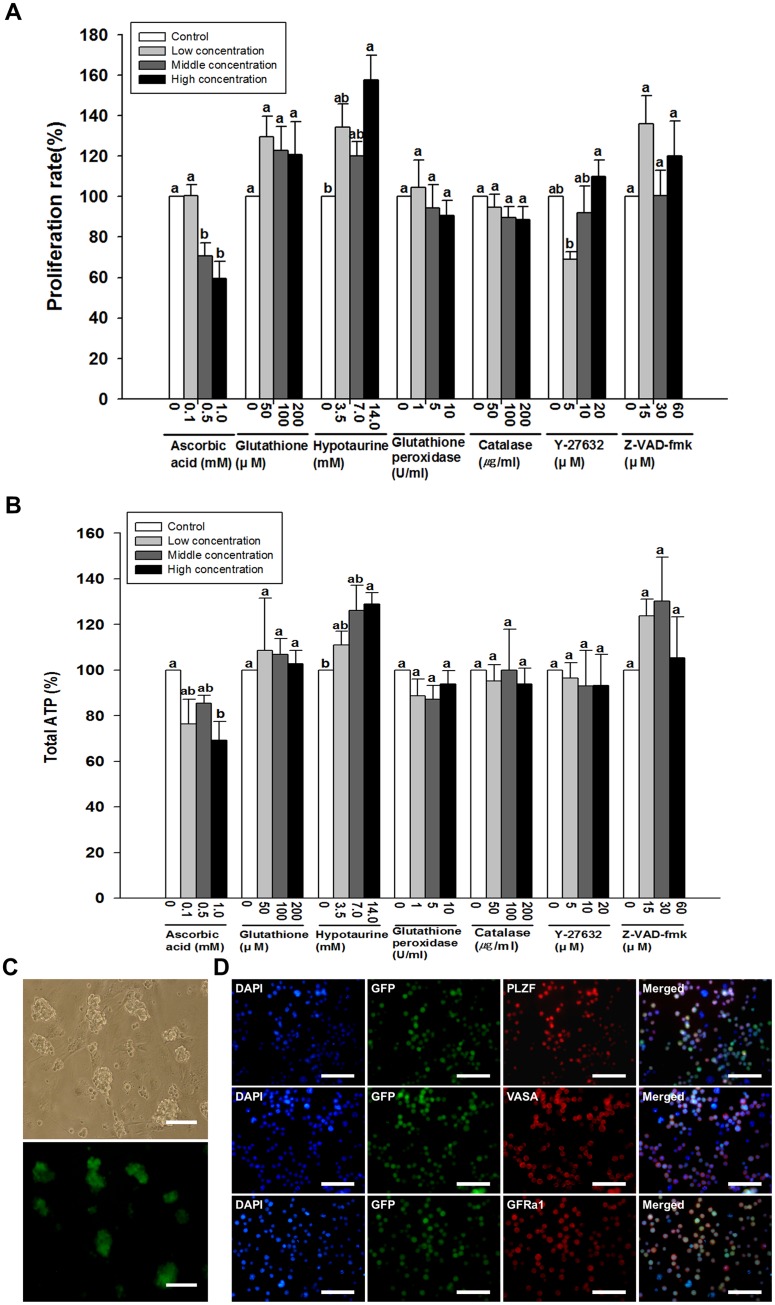
Effect of antioxidants and apoptosis inhibitors on thawed germ cells enriched for SSCs. (A) Effect of antioxidants and apoptosis inhibitors on proliferation of germ cells enriched for SSCs after thawing. (B) Effect of antioxidants and apoptosis inhibitors on ATP production by germ cells enriched for SSCs after cryopreservation. (C-D) Characterization of germ cell colonies after cryopreservation in the presence of 14 mM hypotaurine, thawing and 7 days of culture. (C) Bright/dark-field image. (D) Immunocytochemistry for DAPI (blue), eGFP (green) and PLZF, VASA, or GFRα1 (red). Values are means ± SEM (n = 3 independently established cultures for each treatment). Different letters within each treatment group indicate significant difference (*P* < 0.05) between control and different dosages of each cryoprotectant. Scale bars: (C) = 100 μm; (D) = 75 μm;.

Following cryopreservation for one month, cells were thawed and recovery rates were determined. No significant differences in recovery rate were observed between controls and any treatment (S1 Fig). To determine functionality of thawed germ cells, cells were cultured for one week to determine proliferation capacity. Compared to controls, few treatments had significantly different post-thaw proliferation capacities. The exceptions were cells that were cryopreserved in media containing 0.5 and 1 mM ascorbic acid and 14 mM hypotaurine (Fig 1A). Proliferation capacity of cells cryopreserved with 0.5 and 1 mM ascorbic acid was significantly lower, in a dose dependent manner, compared to control (0.5 mM; 70.8 ± 6.4% and 1.0 mM; 59.6 ± 5.4%; *P* < 0.05). In contrast, the proliferation capacity of cells cryopreserved with 14 mM hypotaurine was significantly greater than control. Interestingly, cells cryopreserved in cryopreservation media containing 3.5 and 7 mM hypotaurine had numerically, however non-significant (*P* > 0.05), higher proliferation capacities than control (3.5 mM; 134.4 ± 11.3%, 7 mM; 119.9 ± 7.2%, and 14 mM; 157.5 ± 12.4%,). In addition to observing recovery rate and proliferation capacity, post thaw germ cell colony morphology was evaluated for cells cryopreserved with 14 mM hypotaurine. After culture for 1 week, most germ cells formed apparently normal germ cell colonies (Fig 1C) containing cells that expressed VASA (a marker of germ cell lineage), PLZF and GFRα1 (markers of undifferentiated spermatogonia including SSCs) (Fig 1D).

### Effects of antioxidants and apoptosis inhibitors on mitochondrial activity of germ cells enriched for SSCs after thawing

To evaluate the mechanism of action of evaluated cryoprotective agents, mitochondrial activity of thawed germ cells was determined. ATP usage was evaluated because previous work has reported that the mechanism of action of hypotaurine as a cryoprotectant is to increase mitochondrial activity, presumably improving viability and integrity of the cell [31, 32]. In the present study, the effects of various cryoprotective agents on mitochondrial activity mirrored the results of the proliferation analysis. Compared to controls, the only cells that had significant differences in ATP production, were cells that were cryopreserved in media containing 1 mM ascorbic acid or 14 mM hypotaurine. As seen in the proliferation assay (Fig 1A), cryopreservation with 1 mM ascorbic acid resulted in significantly lower mitochondrial activity, whereas cryopreservation with 14 mM hypotaurine resulted in significantly greater mitochondrial activity (Fig 1B; *P* < 0.05). Interestingly, cryopreservation with 3.5 and 7 mM hypotaurine resulted in numerical, dose dependent, although non-significant (*P* > 0.05), increases in mitochondrial activity (3.5 mM; 111.0 ± 6.1%, 7 mM; 126.2 ± 10.9%, and 14mM; 128.9 ± 4.9%).

### Effects of combinations of hypotaurine, Z-VAD-fmk and trehalose on cryopreservation of germ cells enriched for SSCs

**Fig 2:**
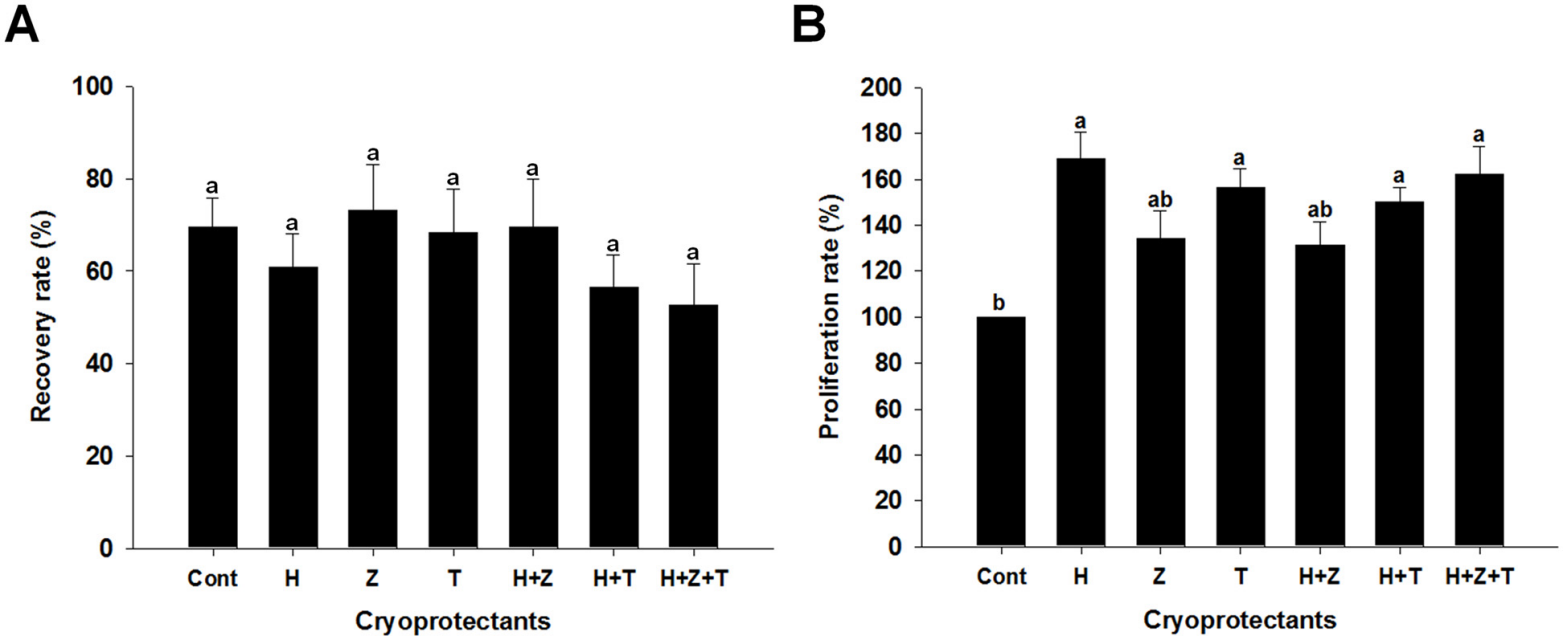
Effects of combinations of hypotaurine, Z-VAD-fmk, and trehalose on recovery rate and proliferation capacity of germ cells enriched for SSCs following cryopreservation. (A) Percentage of viable cells recovered after thawing. (B) Proliferation potential of cells recovered after thawing. Control = basal freezing medium containing 10% DMSO and 10% FBS; H = basal freezing medium with hypotaurine (14 mM); Z = basal freezing medium with Z-VAD-fmk (15 μM), T = basal freezing medium with trehalose 200 mM, H+Z = basal freezing medium with hypotaurine, and Z-VAD-fmk, H+T = basal freezing medium with hypotaurine and trehalose and H+Z+T = basal freezing medium with hypotaurine, Z-VAD-fmk and trehalose. Values are means ± SEM (n = 3 independently established cultures for each treatment). Different letters indicate significant difference (*P* < 0.05) between treatments.

Analyses of recovery, proliferation capacity, and mitochondrial activity indicated that cryopreservation of germ cells enriched for SSCs in the presence of 14 mM hypotaurine is an efficient, effective method for SSC cryopreservation. Previously, it was demonstrated that cryopreservation of germ cells enriched for SSCs in the presence of 200 mM trehalose was also effective for the long-term cryopreservation of murine SSCs [14, 28]. Trehalose, a reducing disaccharide, appears to provide a cryoprotective benefit through dehydration and stabilization of cell membrane proteins [33]. Therefore, different combinations of ACAs were evaluated to determine if combinations would be more effective than individual ACAs on cryopreservation of germ cells enriched for SSCs. Because of the numerical, non-significant improvement in proliferation rate (136.2 ± 13.8%; *P* > 0.05) and ATP production (123.8 ± 7.3%; *P* > 0.05), combinations containing Z-VAD-fmk (15 μM), a cysteine protease and apoptosis inhibitor [34], in addition to trehalose (200 mM) and hypotaurine (14 mM) were also evaluated. Synergistic effects were evaluated by observing the post-thaw recovery and proliferation capacity of germ cells enriched for SSCs that were cryopreserved in the presence of 14 mM hypotaurine (H), 200 mM trehalose (T), or 15μM Z-VAD-fmk (Z) in combination or alone. No significant differences in recovery rate between cells cryopreserved with control basal freezing media or media containing individual or combined cryoprotectants was observed (Fig 2A). In contrast, the proliferation capacity of thawed germ cells enriched for SSCs was significantly greater than control when cells were cryopreserved with 14 mM hypotaurine (168.9 ± 11.4%), 200 mM trehalose (156.3 ± 8.2%), or combinations containing hypotaurine and trehalose (150.1 ± 6.5%) or hypotaurine, trehalose and Z-VAD-fmk (162.0 ± 12.4%; Fig 2B; *P* < 0.05).

### Effects of serum replacement in cryopreservation media on cryopreservation of germ cells enriched for SSCs

**Fig 3:**
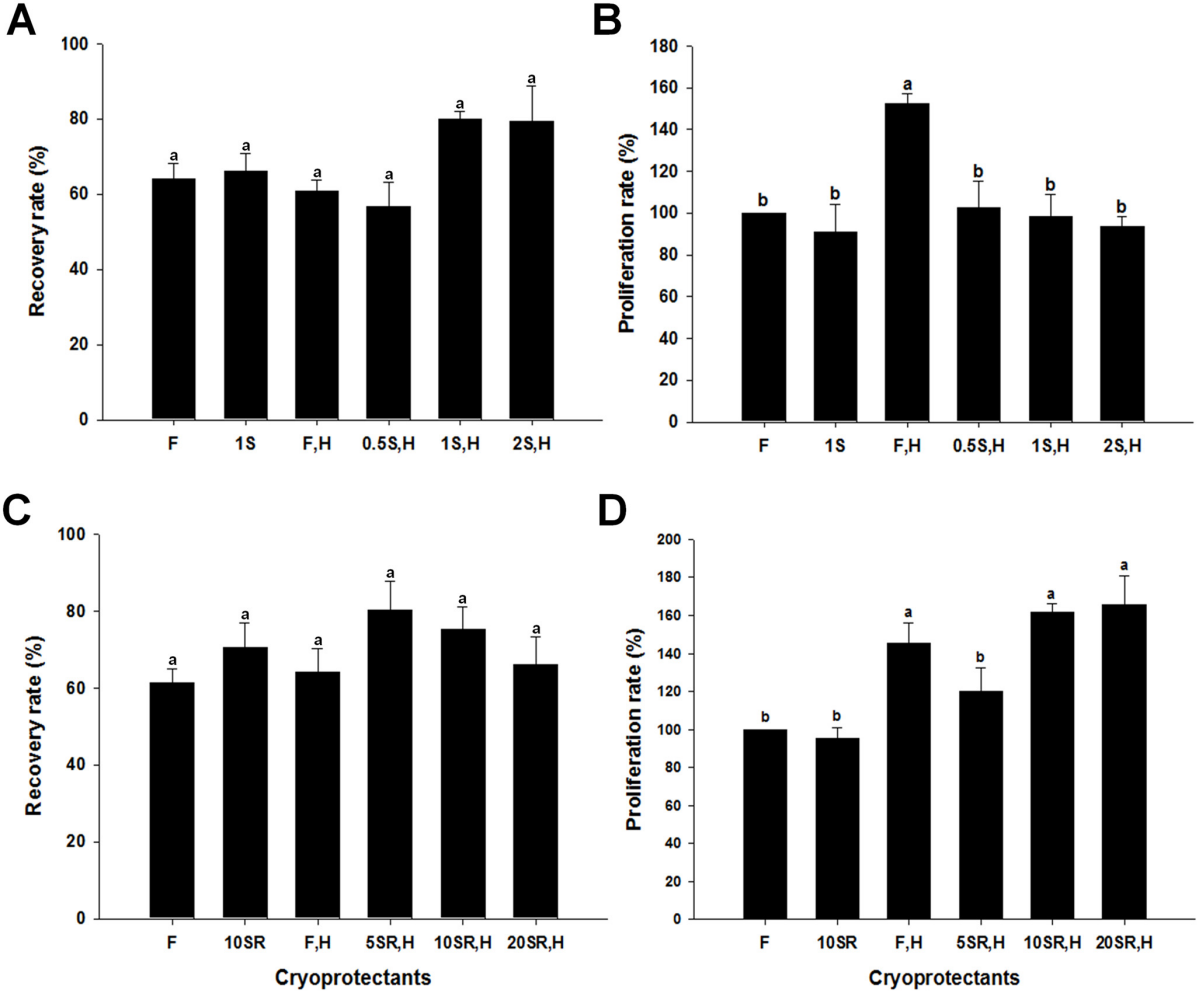
Comparison of FBS to Sericin or KSR as cryoprotectant additives for recovery and proliferation capacity of thawed germ cells enriched for SSCs. (A) Percentage of viable cells recovered after cryopreservation in the presence of FBS or Sericin. (B) Proliferation capacity of cells after cryopreservation in the presence of FBS or Sericin. (C) Percentage of viable cells recovered after cryopreservation in the presence of FBS or KSR. (D) Proliferation capacity of cells after cryopreservation in the presence of FBS or KSR. F = basal freezing medium (containing 10% DMSO) and 10% FBS; 1S = basal freezing medium and 1% Sericin; 10SR = basal freezing medium and 10% KSR; F, H = basal freezing medium with FBS and hypotaurine (14 mM); 0.5, 1, 2S, H = basal freezing medium with 0.5, 1, 2% Sericin and Hypotaurine; 5, 10, 20SR, H = basal freezing medium with 5, 10, 20% KSR and Hypotaurine. Values are means ± SEM (n = 3 independently established cultures for each treatment). Different letters indicate significant difference (*P* < 0.05) between treatments.

Inclusion of FBS in cell culture and cryopreservation media is a common tool used to deliver various nutrients to cells in vitro. However, the lack of defined components in FBS can present problems when using different FBS batches or when attempting to replicate previous research. Thus, the inclusion of Sericin and Knockout Serum Replacement (KSR) as replacements for FBS in cryopreservation media was evaluated. Recently, Sericin has been demonstrated to be an effective serum replacement for the in vitro survival and growth of various mammalian cells, including stem cells from human adipose tissue and germ line stem cells [35–38]. Additionally, KSR, a synthetic serum, has been used as an alternative to FBS in cryopreservation and culture of various stem cells including SSCs [28]. To evaluate the effectiveness of serum-free cryopreservation media, germ cells enriched for SSCs were cryopreserved in media containing 14 mM hypotaurine and either FBS (10%), Sericin (0.5, 1, and 2%) or KSR (5, 10, and 20%). Concentrations of serum replacements were chosen based on results from previous work as described above. No significant differences were observed in recovery rate between thawed cells that were cryopreserved in basal freezing media containing 10% FBS, 1% Sericin, 10% FBS + 14 mM hypotaurine or 0.5, 1 or 2% Sericin +14 mM hypotaurine (Fig 3A). Interestingly, thawed cells that were cryopreserved in basal freezing media containing 10% FBS and 14 mM hypotaurine had significantly greater proliferation capacity than cells frozen with media containing 10% FBS alone, 1% Sericin, or 0.5, 1 or 2% Sericin +14 mM hypotaurine (Fig 3B). Similar results were observed with KSR serum replacement. No significant differences were observed in recovery rates between thawed cells that were cryopreserved in basal freezing media containing 10% FBS, 10% KSR, 10% FBS + 14 mM hypotaurine or 5, 10 or 20% KSR +14 mM hypotaurine (Fig 3C). Interestingly, thawed cells that were cryopreserved in basal freezing media containing 10% FBS, 10% KSR or 20% KSR with 14 mM hypotaurine had significantly greater proliferation capacity than cells frozen with media containing 10% FBS alone, 10% KSR alone, or 5% KSR +14 mM hypotaurine (Fig 3D). Collectively, these data indicate that KSR can replace FBS in germ cell cryopreservation media and that the synergistic effect of FBS and hypotaurine is also evident when hypotaurine is combined with KSR in cryopreservation media.

### Effects of cryopreservation media containing hypotaurine on SSC function

**Fig 4:**
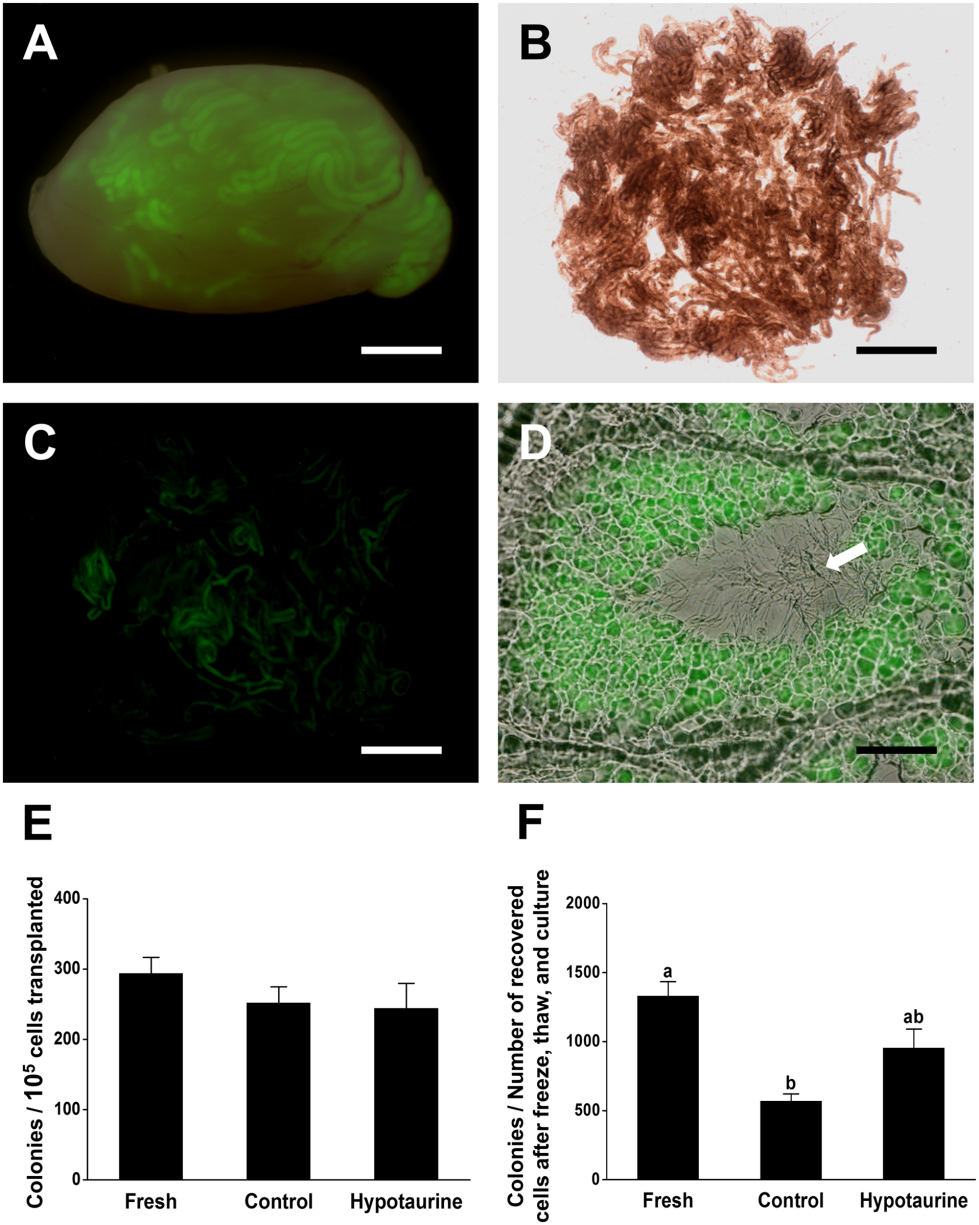
Functional SSC activity was demonstrated by counting the number of donor derived colonies after transplantation. (A) Dark-field fluorescence image of a recipient testis transplanted with germ cells cryopreserved in the presence of 14 mM hypotaurine. Colonies of donor spermatogenesis are distinct green regions of the recipient seminiferous tubules. (B, C) Dispersed seminiferous tubules from a recipient testis. Bright field (B). Dark field (C). (D) Cryosection of donor-derived germ cell colonies. Complete spermatogenesis is illustrated by the presence of sperm (white arrow) in the lumen of the seminiferous tubule. (E) The number of colonies per 10^5^ transplanted cells. Fresh: non-cryopreserved cells (294.0 ± 22.6), Control: cells cryopreserved in basal freezing media (252.0 ± 22.6), and Hypotaurine: cells cryopreserved in basal freezing media with 14 mM hypotaurine (244.0 ± 35.2). (F) The number of colonies per 10^5^ transplanted cells normalized for recovery rate and proliferation capacity. Fresh (1332.0 ± 103.0), Control (572.0 ± 51.3), and Hypotaurine (953.0 ± 137.0) (n = 2 experiments per treatment; total number of mice/testes analyzed were 10/11, 9/12, and 9/16 for fresh, control, and hypotaurine, respectively). Scale bars: (A) = 2 mm; (B, C) = 4 mm; (D) = 50 μm.

To definitively evaluate the effects of cryopreservation media on SSCs specifically, thawed, cultured, germ cells were transplanted into recipient testes. Germ cells enriched for SSCs that were cryopreserved in basal freezing media containing 14 mM hypotaurine were thawed and cultured for 7 days. Following culture, cells were transplanted into recipient testes (Fig 4). Two months after transplantation, testes were removed and evaluated for colonization. The presence of eGFP expressing colonies of spermatogenesis indicated the functional capacity of cryopreserved SSCs (Fig 4A–4C). Furthermore, histological analysis demonstrated the ability of the cryopreserved transplanted SSCs to facilitate complete spermatogenesis (Fig 4D). After quantification, no significant differences in the number of colonies per 10^5^ germ cells transplanted were observed between cells that had not been cryopreserved (294.0 ± 22.6), cells that had been cryopreserved in basal freezing media (252.0 ± 22.6), and cells that had been cryopreserved in basal freezing media containing 14 mM hypotaurine (244.0 ± 35.2; Fig 4E). To account for differences in post-thaw culture dynamics between cells frozen in basal freezing media and basal freezing media containing 14 mM hypotaurine, colony number was normalized to the number of recovered cells after freezing, thawing and culture. As expected, significantly more normalized colonies were observed in testes transplanted with fresh SSCs compared to testes transplanted with post-thaw SSCs that were cryopreserved in basal freezing media. Importantly, no significant difference was observed between the number of normalized colonies formed by germ cell cultures established with fresh SSCs or cultures established from SSCs that were cryopreserved in basal freezing media containing 14 mM hypotaurine (Fig 4F). This data indicates that although cryopreservation in basal freezing media results in fewer colonies of spermatogenesis compared to fresh cells, the addition of 14 mM hypotaurine to basal freezing media abolishes the deleterious effect of the freezing process on the functional capacity of the SSC population.

## Discussion

The objective of the present study was to evaluate the effectiveness of various cryopreservation media additives, including antioxidants and apoptosis inhibitors, on the recovery, proliferation capacity, and mitochondrial activity of thawed germ cells enriched for SSCs. To enrich for SSCs, testis cells were sorted by MACS using anti-Thy-1 microbeads as previously described [27]. Following cryopreservation for 1 month in various cryopreservation media, thawed cells were evaluated for recovery rate, proliferation capacity, and mitochondrial activity. Antioxidants and apoptosis inhibitors used as cryopreservation additives were chosen based on previous reports demonstrating their beneficial use in reducing the formation of reactive oxygen species and in prevention of mitochondrial dysfunction and apoptosis [18–20, 22–24].

Regardless of concentration or additive, no differences in recovery rate, based on post-thaw viability, between treatments were observed. This data suggests that germ cells enriched for SSCs can faithfully withstand the rigors of cryopreservation; however, it is possible that cryodamage may elicit delayed apoptosis that would not be detected using trypan blue exclusion at the time of thawing. Thus, after thawing, cells were evaluated for mitochondrial activity and cultured for one week to evaluate proliferation capacity. Interestingly, cells that were cryopreserved in basal freezing media containing 14 mM hypotaurine had significantly greater post-thaw mitochondrial activity and proliferation capacity than cells that were cryopreserved in basal freezing media alone (Fig 1; *P* < 0.05). Hypotaurine is the final metabolite of cysteine metabolism and is a precursor of Taurine [39]. Additionally, hypotaurine is a strong antioxidant that has the capacity to remove free hydroxyl radicals from the body [40]. Although mitochondrial activity is low in SSCs [41], changes in mitochondrial activity could result in differential proliferation capacities. Most cellular free radicals are formed in the mitochondria and although some reactive oxygen is needed for cellular signaling, various physiological processes and the stress response, excess free radical formation can be deleterious to cellular function. Therefore, through the control of oxidative stress caused by free radicals during cryopreservation or thawing, post-thaw mitochondria activity, thus proliferation capacity, is improved if hypotaurine is included in the cryopreservation media. Interestingly, similar effects were not observed with other antioxidants such as catalase and glutathione peroxidase. This may be due to decreased function of these enzymes during the freezing process. Future studies will evaluate post-thaw catalase and glutathione peroxidase activity in addition to examining the effects of higher enzyme concentrations and enclosure of the enzymes in a protective liposome. Furthermore, more detailed analyses of the mechanisms of apoptosis inhibition, including evaluation of caspase activity or DNA fragmentation, could provide more evidence of the mechanism of action of particular cryopreservation agents with regards to SSC survival.

Previously, it was demonstrated that addition of trehalose to cryopreservation media proved beneficial to the post-thaw survival of germ cells enriched for SSCs [14, 28]. Trehalose is a non-reducing disaccharide which is believed to facilitate cryopreservation by preventing ice crystal formation and/or stabilizing proteins within the plasma membrane [33]. Indeed, others have reported that cryopreservation of various cells, including hematopoietic cells and mammalian germ cells, in media containing trehalose enhances survival rate and colony formation ability [42–44]. In addition to trehalose, inclusion of FBS and DMSO in cryopreservation media has proven beneficial for post thaw survival and function of various stem cells [14, 22, 23, 28]. Z-VAD-fmk is a chemical that has been shown to prevent apoptosis by inhibiting caspase cysteine proteases [34]. Because of the ability of Z-VAD-fmk to prevent apoptosis, it was hypothesized that its inclusion in cryopreservation media would be beneficial for germ cell cryopreservation. However, no significant improvement was observed compared to control when germ cells enriched for SSCs were cryopreserved in media containing Z-VAD-fmk (*P* < 0.05). Nevertheless, inclusion of 15 μM of Z-VAD-fmk into germ cell cryopreservation media did numerically improve germ cell proliferation capacity (136.2 ± 13.8%) over control. Because of the importance of cryopreservation for various stem cell techniques, including banking and transplantation, a more thorough examination of the ability of Z-VAD-fmk (and other apoptosis inhibitors) to improve SSC cryopreservation is warranted. Future analyses could include examination of additional cryoprotectant agents or concentrations as well as additional methods of apoptosis analysis.

Because of the ability of trehalose and hypotaurine to improve the cryopreservation efficiency of germ cells enriched for SSCs, through presumably different mechanisms, it was hypothesized that a combination of both would provide additional benefit. Moreover, due to the numerical improvement of germ cell function following cryopreservation with Z-VAD-fmk, coupled with its anti-apoptosis function, it was hypothesized that its combination with trehalose, hypotaurine, or both, would also provide further additional benefit. Compared to control, as expected, cryopreservation of germ cells enriched for SSCs in the presence of hypotaurine or trehalose, but not Z-VAD-fmk significantly improved post thaw proliferation capacity. When combined, cryopreservation of germ cells in the presence of hypotaurine and trehalose and hypotaurine, trehalose, and Z-VAD-fmk, but not hypotaurine and Z-VAD-fmk significantly improved post thaw proliferation capacity. Interestingly, no synergistic effects were observed as no significant difference was observed in proliferation capacity of thawed cells that were cryopreserved in the presence of hypotaurine alone, trehalose alone, hypotaurine and trehalose, or hypotaurine, trehalose and Z-VAD-fmk.

Previous work has shown that serum is a useful additive for increasing the cryopreservation efficiency of various cells and tissues [45, 46]. However, serum is not suitable for human clinical applications because of its lack of definition and potential to carry harmful agents such as viruses [12, 47]. The present study investigated the efficacy of replacing FBS in the basal freezing media or basal freezing media containing 14 mM hypotaurine, with KSR or Sericin. Inclusion of KSR, rather than FBS, in cryopreservation media containing trehalose was demonstrated to be effective in maintaining the proliferation capacity of SSCs after thawing [28]. Replacement of FBS in basal freezing media with either Sericin or KSR did not significantly impact the recovery rate or proliferation capacity of thawed germ cells enriched for SSCs. Interestingly, the synergistic effect of hypotaurine on the efficiency of cryopreservation that was seen with basal media containing FBS was observed when FBS was replaced with KSR, but not Sericin. Furthermore, there was no difference in proliferation capacity of thawed germ cells enriched for SSCs regardless of if the cells were cryopreserved in basal freezing media contain 10% FBS and 14 mM hypotaurine or freezing media containing 10 or 20% KSR and 14 mM hypotaurine. KSR has been used as a serum replacement for the culture of embryonic stem cells [48]. Importantly, KSR did not alter the undifferentiated state of the embryonic stem cells, a characteristic that is also essential for the culture and preservation of SSCs.

In conclusion the data have demonstrated that inclusion of 14 mM hypotaurine in basal freezing media containing DMSO and either FBS or KSR significantly improves the post thaw mitochondrial activity and proliferation capacity of germ cells enriched for SSCs. Furthermore, the ability of thawed SSCs to form donor derived colonies of spermatogenesis was not different than non-cryopreserved cells. Finally, the feasibility of replacing FBS with KSR to generate a serum free cryopreservation medium is of particular importance for the development of similar applications in human clinical settings.

## Supporting Information

S1 Fig Effect of antioxidants and apoptosis inhibitors on recovery rate of germ cells enriched for SSCs after cryopreservation. Different letters within each treatment group indicate significant differences (P < 0.05) between control and different dosages of each cryoprotectant. (Bars: mean ± SEM; n = 4, *P* < 0.05).
(TIF)
