# Supplementary figures and images for: Effect of Antioxidants and Apoptosis Inhibitors on Cryopreservation of Murine Germ Cells Enriched for Spermatogonial Stem Cells
Source: PLoS One. 2016 Aug 22;11(8):e0161372. doi: 10.1371/journal.pone.0161372 (PMC4993461; doi:10.1371/journal.pone.0161372)

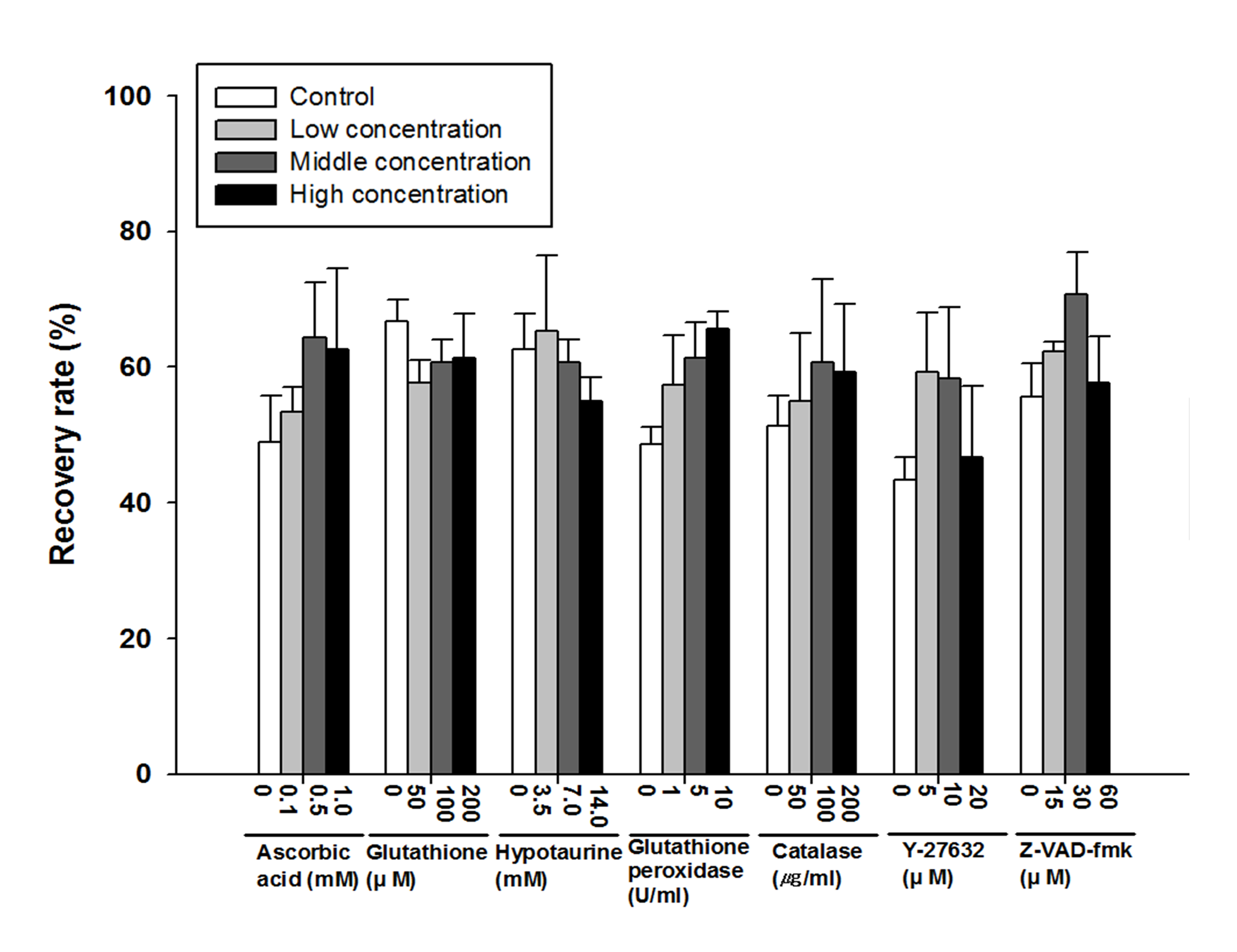

Supplement: S1 Fig — Different letters within each treatment group indicate significant differences (P < 0.05) between control and different dosages of each cryoprotectant. (Bars: mean ± SEM; n = 4, P < 0.05). (TIF) [file pone.0161372.s001.tif]
